# Tat-SF1 Is Not Required for Tat Transactivation but Does Regulate the Relative Levels of Unspliced and Spliced HIV-1 RNAs

**DOI:** 10.1371/journal.pone.0005710

**Published:** 2009-05-27

**Authors:** Heather B. Miller, Kevin O. Saunders, Georgia D. Tomaras, Mariano A. Garcia-Blanco

**Affiliations:** 1 Department of Molecular Genetics and Microbiology, Duke University Medical Center, Durham, North Carolina, United States of America; 2 Center for RNA Biology, Duke University Medical Center, Durham, North Carolina, United States of America; 3 Department of Surgery, Duke University Medical Center, Durham, North Carolina, United States of America; 4 Department of Medicine, Duke University Medical Center, Durham, North Carolina, United States of America; Institut Pasteur, France

## Abstract

**Background:**

HIV-1 relies on several host proteins for productive viral transcription. HIV-1 Tat-specific factor 1 (Tat-SF1) is among these cofactors that were identified by *in vitro* reconstituted transcription reactions with immunodepleted nuclear extracts. At the onset of this work, the prevailing hypothesis was that Tat-SF1 was a required cofactor for the viral regulatory protein, Tat; however, this had not previously been formally tested *in vivo*.

**Methodology/Principal Findings:**

To directly address the involvement of Tat-SF1 in HIV-1 gene expression, we depleted Tat-SF1 in HeLa cells by conventional expression of shRNAs and in T- Rex -293 cells containing tetracycline-inducible shRNAs targeting Tat-SF1. We achieved efficient depletion of Tat-SF1 and demonstrated that this did not affect cell viability. HIV-1 infectivity decreased in Tat-SF1-depleted cells, but only when multiple rounds of infection occurred. Neither Tat-dependent nor basal transcription from the HIV-1 LTR was affected by Tat-SF1 depletion, suggesting that the decrease in infectivity was due to a deficiency at a later step in the viral lifecycle. Finally, Tat-SF1 depletion resulted in an increase in the ratio of unspliced to spliced viral transcripts.

**Conclusions/Significance:**

Tat-SF1 is not required for regulating HIV-1 transcription, but is required for maintaining the ratios of different classes of HIV-1 transcripts. These new findings highlight a novel, post-transcriptional role for Tat-SF1 in the HIV-1 life cycle.

## Introduction

The human immunodeficiency virus type 1 (HIV-1), like all other complex retroviruses, tightly regulates transcription from its genome. This regulation is mediated by both viral and cellular factors [1], [2], [3], [4]. The viral regulatory protein, Tat, stimulates transcription elongation of HIV-1 through a series of events termed Tat transactivation [5], [6], [7], [8], [9], [10], [11], [12]. Tat recruits the human positive transcription elongation factor b (P-TEFb) to the TAR RNA element at the 5′ end of nascent transcripts [2], [5]. Tat interacts directly with cyclin T1 (CCNT1), a component of P-TEFb, which allows recognition of TAR [6]. P-TEFb recruitment has been proposed to be necessary and sufficient for transcriptional elongation [13]. The CDK9 kinase activity of P-TEFb results in hyperphosphorylation of the carboxyl-terminus domain (CTD) of the largest subunit of RNA Polymerase II (RNAPII), leading to efficient elongation [7], [8], [9], [10], [14], [15].

Many groups have investigated the mechanism by which HIV-1 utilizes P-TEFb as a cellular cofactor for Tat transactivation. These studies suggest that P-TEFb is part of a multiprotein complex that associates with RNAPII at the HIV-1 promoter and that other cellular factors also assist in transactivation [16], [17], [18]. Previous studies have used nuclear extract fractions from Tat affinity columns to reconstitute Tat transactivation *in vitro* [16], [18], [19], [20]. One of these studies identified a cellular activity that was required for Tat-specific, TAR-dependent activation of HIV-1 transcription *in vitro*, and it was termed Tat stimulatory factor (Tat-SF) [18], [21]. Further affinity purification of this activity identified a novel, 140-kDa protein that was sequenced and named Tat-SF1 (accession number NP_055315; HUGO gene name *HTAT-SF1*). Immunodepletion of this protein from nuclear extracts resulted in a reduction in Tat transactivation [16], [18], [19], and overexpression of Tat-SF1 resulted in a small increase in Tat transactivation [16]. The increase in Tat transactivation, however, was primarily due to a decrease in basal transcription, and only a small increase in Tat-dependent transcription [16]. In addition to the usual caveats of overexpression data, this result was not recapitulated by the same group when using a different plasmid system [22]. These studies, which used the best technology available at the time, justifiably concluded that Tat-SF1 was a likely cofactor for Tat transactivation *in vitro* and *in vivo*.

Tat-SF1 has also been proposed to be a general elongation factor [18], [19]. It can associate with Tat:P-TEFb transcription elongation complexes in nuclear extracts [14] through a direct CCNT1 interaction [14], [23]. Two other transcription elongation factors, hSPT5 and the RAP30 protein of TFIIF, associate with Tat-SF1 [22]. Tat-SF1 has been shown to be a component of an RNAPII-containing complex that also contains other HIV-1 cellular cofactors such as P-TEFb and hSPT5, and these factors were shown to be recruited to the HIV-1 promoter in HeLa nuclear extract [18]. In a separate study, immunoprecipitation experiments showed that Tat-SF1, along with P-TEFb, TCERG1 (CA150), and TFIIF all associate in an RNAPII-containing complex [24].

In addition to the associations with transcription factors, Tat-SF1 has also been found to interact with several components of the spliceosome. Large RNAP II-containing complexes that associate with 5′-splice sites contain Tat-SF1 [25]. Tat-SF1 also interacts with snRNP proteins U1 70 K, U2B″, and Sm proteins B and B′. In addition, Tat-SF1 associates with all five spliceosomal U snRNAs, and this interaction depends on its RNA recognition motifs (RRMs) [26]. Moreover, the yeast homologue of Tat-SF1, CUS2, helps refold U2 snRNAs to aid in prespliceosome assembly [27]. The association with both elongation and splicing factors has led to the suggestion that Tat-SF1 can couple these two processes [26]. Indeed, another transcription-splicing coupling factor, TCERG1, binds Tat-SF1 directly through multiple interactions with FF domains in the former [28].

Insight into the role of Tat-SF1 in the HIV-1 lifecycle has previously been limited to immunodepletions and *in vitro* analyses or transient overexpression experiments. In this manuscript, we present studies that utilize RNA interference (RNAi) to reevaluate Tat-SF1's role in Tat transactivation and HIV-1 replication *in vivo*. We found that Tat-SF1 depletion did not affect transcription from the HIV-1 LTR and did not alter the overall level of viral transcripts; however, Tat-SF1 depletion resulted in a significant decrease in viral replication. This study demonstrates that the major effect upon knockdown of Tat-SF1 was a change in the ratio of unspliced to fully spliced HIV-1 RNAs. Based on our data, we propose a novel activity for Tat-SF1 as a post-transcriptional regulator of viral pre-mRNAs.

## Materials and Methods

### shRNA constructs

Two different short hairpin RNA (shRNA) sequences targeting Tat-SF1 transcripts in the pSuper vector backbone (OligoEngine) were gifts from Dr. Bryan Cullen (Duke University). Tat-SF1(A) consists of a hairpin targeting the following Tat-SF1 sequence: GAGTCAGATGACAAGGAAG. Tat-SF1(B) targets GAGAATCTGTGGAACTTGC. A third shRNA targeting Tat-SF1, Tat-SF1(C), was expressed from the pSM2 vector (Open Biosystems), obtained from the Duke RNAi Facility. Tat-SF1(C) targets the following Tat-SF1 sequence: GGCCTTCTAGAGCAAGGCATTT. The GFP targeting and non-silencing control shRNAs were also in pSM2. Empty pSuper, used as a negative control in transient knockdowns, was a gift from Dr. Vann Bennett (Duke University). To create tetracycline inducible shRNA constructs, hairpin sequences were subcloned from pSuper by digestion with BamH1 and XhoI and ligated into the corresponding sites in pcDNA5/FRT/TO (Invitrogen). Hairpin sequences targeting GFP and Tat-SF1 in the pSM2 vector were PCR amplified using the following oligonucleotides: 5′-CCA TGG GGA TCC CAG CAC ATA TAC TAG TCG AC 3′ AND 5′ CCA TGC GGC CGC TAA TTC AGC TTT GTA AAA ATG- 3′. Gel-purified PCR products were digested with BamH1 and NotI and ligated into the corresponding sites in pcDNA5/FRT/TO. The structure of all constructs was verified by DNA sequence analysis.

### Plasmids

The HIV-CAT reporter and the CMV-driven Tat plasmid, pcTat have been described previously [29], [30]. The SV40-driven luciferase reporter, pGL3-Control Vector (Promega), was used to control for transfection efficiency. The HIV-1 proviral indicator plasmid, pNL-Luc-HXB was described in [31]. The HIV-1 pNL4-3 plasmid, pSG3ΔEnv, was described in [32]. pSG3ΔEnv contains a four nucleotide insertion mutation that encodes a truncated gp120, without disrupting any of the known splicing regulatory *cis* elements. The plasmid expressing the VSV-G envelope, pHIT/G was described in [33]. The plasmid expressing the AMLV envelope, pSV-A-MLV-env was described in [34].

### Cell culture

The Flp-In ™ T- Rex™ -293 cell line is a stable, tetracycline-inducible, mammalian expression cell line. It was used here to express shRNAs under the control of a CMV promoter that contained two tandem repeats of the *tet* operator 2 sequence. T-Rex-293 cells that harbored the pFRT/lacZeo and pcDNA6/TR plasmids were cultured in DMEM (high glucose) supplemented with 10% fetal bovine serum, 1% penicillin/streptomycin and 1% L-glutamine. Zeocin (100 µg/mL) and blasticidin (15 µg/mL) were included to select for cells containing a single integrated FRT site and the Tet repressor plasmid. A GFP-expressing plasmid, Gint [35], was linearized with DraIII and transfected into T-Rex-293 cells using Lipofectamine 2000 (Invitrogen). Genetecin (500 µg/mL) was added to culture media and was changed every three days until a stable, polyclonal population was obtained. These cells were sorted by fluorescence activated cell sorting (FACS) to isolate the population with high GFP expression. shRNAs described above in pcDNA5/FRT/TO and empty vector pcDNA5/FRT/TO were each cotransfected with pOG44 (to express Flp recombinase) into GFP-positive T-Rex -293 cells, and stably transfected cells were selected with hygromycin (200 µg/mL), blasticidin (15 µg/mL), and geneticin (500 µg/mL). Tetracycline-reduced FBS (Hyclone) was used for all cultures that contained a tetracycline-inducible plasmid to minimize basal shRNA expression. Tetracycline concentrations and induction times were optimized for each shRNA expressed to achieve maximal knockdown. Cell viability was assessed at various time pointstime points after shRNA induction using trypan blue exclusion. The dye was added to trypsinized cells to 0.2% (v/v), and viable cells were counted on a hemocytometer. Cell confluency was evaluated at various time points by imaging cells on an Olympus (Melville, NY) IX71 epifluorescence microscope. Images were acquired with an Olympus DP70 digital camera, and images were processed with DP Controller software (Olympus).

HeLa cells were cultured in DMEM (high glucose) with 10% FBS and 1% penicillin/streptomycin. HeLa cells stably expressing the non-silencing shRNA or Tat-SF1(C) were created by linearizing the plasmids with BstBI and ApaI, respectively, before transfection into HeLa cells using Lipofectamine 2000. Positive transfectants were selected with puromycin. 293T and TZM-bl cells [36] were cultured in DMEM (high glucose) with 10% FBS and 1% penicillin/streptomycin.

### Western blot analysis

Protein concentrations of cell lysates were determined by a Bradford assay (Bio-Rad) and 30 µg of protein was separated on SDS-PAGE gels before transferring to PVDF membranes (BioRad). The membranes were probed with rabbit polyclonal antibodies to CCNT1 (AbCam) at a 1∶500 dilution, PTB (Intronn, LLC, Durham, NC) at a 1∶5000 dilution or antiserum to Tat-SF1 (Research Genetics Inc, Huntsville, AL) at a 1∶500 dilution. Rabbit secondary antibody (Amersham) was used at a 1∶5000 dilution and proteins were detected with ECL (Amersham) or SuperSignal West Chemiluminescence Substrate (Pierce).

### Pseudotyped virus production and HIV-1 replication assays

293T cells were plated in 100-mm dishes and cotransfected with 1 µg pHIT/G (for VSV-G envelope production) or 1 µg pSV-A-MLV-env (for AMLV envelope production) and 10 µg pNL-Luc-HXB using the calcium phosphate method. Media was changed the following day and supernatants were passed through a 0.45-µm pore size filter 48 hours post transfection. Viral supernatants not used immediately were frozen at −80°C.

T-Rex-293 cell lines expressing either empty pcDNA5/FRT/TO or a plasmid with an shRNA targeting GFP or Tat-SF1 were induced with 2.5 µg/mL tetracycline for 48 hours. Then, 2×10^5^ cells were plated per well of a 6-well plate, keeping tetracycline present in the media. Cells were imaged in phase contrast and fluorescence before and after infection to confirm equal confluency. 72 hours post induction, media was removed and 2 mL of viral supernatant was added to the cells. HeLa cells stably expressing either the non-silencing shRNA or an shRNA targeting Tat-SF1 were infected in the same manner. Since 293 and HeLa cells do not express the HIV-1 specific CD4 receptor, only VSV-G or AMLV pseudotyped viruses were able to infect the target cells. After 24 or 48 hours, a luciferase assay was performed on the cell lysates. All values were normalized to the protein concentration of the lysate, determined by the BCA Protein Assay (Pierce).

### TZM-bl assays of infectivity

TZM-bl cells were used for replication-competent HIV-1 infection because they express endogenous CXCR4, transgenic CD4 and CCR5, and integrated Tat-dependent beta-galactosidase and luciferase reporter genes. They have also been shown to be susceptible to RNAi [37]. Empty vector, a non-silencing shRNA, or Tat-SF1-targeting shRNAs were co-transfected with a GFP-expressing plasmid into TZM-bl cells using FuGene6 (Roche). Forty-eight hours post-transfection, GFP-positive cells were collected by FACS. 8×10^3^ sorted cells were plated per well in a 96-well plate in DMEM supplemented with DEAE (15 ng/µL). For reverse transcriptase assays, 1.6×10^4^ sorted cells were plated per well in a 12-well plate. Plated cells were infected with replication-competent HIV-1_TT31_, HIV-1_JRFL_, or HIV-1_IIIB_ at multiplicities of infections of 0.004, 0.02, or 0.05 respectively. HIV-1_TT31_ is a chimeric virus composed of the *env* gene from an early-transmitted primary virus isolate [38] and the genome of HIV-1_NL4-3_. HIV-1_TT31_ was constructed by cotransfecting 293T cells with a single-genome-amplified *env* gene from patient TT31, and the recombinant HIV_NL4-3_ backbone, pHIVenvBstEII*nef-hisD* [39]. Supernatants from the transfected cells were harvested 3 days post-transfection and used to generate viral stocks in peripheral blood mononuclear cells. At 3 or 6 days post-infection with replication-competent virus, lysates were prepared for luciferase assays with the Britelite Gene Assay System (PerkinElmer) according to the manufacturer's instructions. Luciferase values were read on a Victor^3^ (PerkinElmer) reader. Values shown are the means of three independently transfected, FACS sorted, and infected wells. The error bars represent standard error. Lysates from GFP-sorted, uninfected cells were prepared in SDS lysis buffer to analyze levels of Tat-SF1 by western blot both at the time of infection (48 hours after knockdown) and at the end of the experiment.

### Reverse transcriptase assays of infectivity

Virion-associated reverse transcriptase was measured as described previously [40], [41]. Briefly, cell culture supernatants from cells infected with HIV-1_IIIB_ were harvested by centrifugation for 5 min at 1500 rpm and treated with Triton X-100 at a final concentration of 1%. 10 µL of treated supernatant was added to 40 µl of reaction cocktail containing: 50 mM Tris-HCl pH 7.8, 75 mM KCl, 2 mM Dithiothreital, 5 mM MgCl_2_, 5 µg/mL Poly A, 0.03 units/mL oligo dT_12–18_, 0.05% Igepal CA-630, 10 mM EGTA, and 10 µCi/mL dTTP [^32^P] (MP Biomedical, Solon,OH). Reactions were incubated at 37°C for 90 min. 40 µL of the reverse transcription reaction was blotted onto DE 81 membrane (Thermal Scientific, Odessa, TX) using a Schleicher and Schuell Minifold and a vacuum. The blotted membranes were washed briefly with 2× SSC (0.3 M NaCl, 0.03 M Sodium Citrate) at room temperature and exposed to a storage phosphor screen overnight. The screen was scanned with a Typhoon phosphor image, and the data analyzed with ImageQuant 5.2 software (GE Healthcare, Piscataway, NJ).

### Tat transactivation assays

HeLa cells were seeded in a 24-well plate before transient transfection with 0.4 µg of either empty pSuper plasmid, a non-silencing shRNA , or Tat-SF1(A) and Tat-SF1(B) in combination using Lipofectamine 2000. After 24 hours of knockdown, cells were passaged to a 6-well plate before transient transfection of reporter plasmids. T-Rex-293 cells were induced with 2.5 µg/mL tetracycline for 48 hrs and seeded in a 6-well plate before transient transfection of reporter plasmids. The calcium phosphate method was used to transfect 0.5 µg of HIV-CAT, 50 ng of SV40-LUC as an internal control for transfection efficiency, and the indicated amount of either pcTat or a CMV-driven empty vector. Twenty-four hours later, lysates were prepared using 500 µL of Cell Culture Lysis Reagent (Promega). Luciferase assays were performed with 40 µL of lysate using a Luciferase Assay Kit (Promega) according to the manufacturer's instructions. Readings were obtained using a Lumat LB 9507 Luminometer (EG&G Berthold). CAT assays were performed using the diffusion method on the same volume of lysate [42]. Both reporter gene assays were background corrected with values obtained from lysis buffer alone. All CAT values were normalized to luciferase values.

### Northern blot analysis

T-Rex-293 cells were induced with 2.5 µg/mL of tetracycline for 48 hours, plated in 6-well plates (4×10^5^ cells/well), and after an additional 24 hours, transiently transfected with 1 µg of pSG3ΔEnv by the calcium phosphate method. Each shRNA-expressing cell line was transfected in triplicate and tetracycline was present in the media at all times. Media was replaced the day following transfection, and 48 hours post-transfection, cells were washed and total RNA was extracted with TRIzol (Invitrogen). 4 µg of total RNA was subjected to a glyoxylation reaction for 1 hour at 55°C. RNAs were immediately chilled for 10 minutes on ice before separation on a 0.8% agarose gel. RNA was transferred overnight to BrightStar-Plus positively charged nylon membrane (Ambion) by capillary action in 10× SSC. After crosslinking the membrane in a UV Stratalinker 2400 (Stratagene), transfer efficiency was assessed by staining the membrane with methylene blue and the agarose gel with ethidium bromide. Membranes were prehybridized for 2 hours in 0.5 M Na_3_PO_4_, pH 7.2, 7% (w/v) SDS, 1 mM EDTA, pH 7.0 at 68°C. An HIV-1 LTR radiolabelled probe was generated from a 25 ng KpnI-HindIII fragment of the HIV-1 LTR described in [43] using [α-^32^P]dCTP and the Random Primers DNA Labeling Kit (Invitrogen). Denatured probe (>1×10^7^ cpm) was added to hybridization solution and incubated with the membrane for at least 1 hour at 68°C. Membranes were washed once at room temperature in 1× SSC+0.1% SDS, then three times at 68°C in 0.5× SSC+0.1% SDS, followed by overnight exposure to film with an intensifying screen at −80°C or a phosphor screen. Membranes were stripped with boiling 0.5% SDS and reprobed with a random primed GAPDH PCR product. Northern blots were quantified with ImageQuant by first background correcting each band. The proportion of each of the three HIV-1 RNA classes in cells depleted of Tat-SF1 was expressed relative to the proportion of the same RNA class in cells treated with the control shRNA. Quantification of total HIV-1 transcripts levels was achieved by adding the values of the 9 kb, 4 kb, and 2 kb signals and normalizing to the GAPDH signal. Statistical significance was determined using a paired Student's t test, and significance was set at p<0.05.

### Reverse transcription and real-time PCR detection of HIV-1 transcripts

Total cellular RNA was isolated using TRIzol per the manufacturer's protocol. RNA samples were then subjected to two rounds of DNase digestion to remove residual DNA using DNA-free (Ambion). To verify the removal of DNA, each RNA sample was tested in triplicate by real-time PCR using SYBR Green PCR Master Mix (Applied Biosystems) and HIV-1 LTR specific primers (HIVshortF and HIVshortR) described elsewhere [44]. Real-time reactions were performed in an ABI 7500 Real-time PCR System (Applied Biosystems) with the following cycling profile: one cycle of 50°C for 5 min, one cycle of 95°C for 10 min, 40 cycles of 95°C for 15 s and 60°C for 1 min. Reverse transcription was performed on 300 ng of each RNA sample for 1 h at 37°C using M-MLV Reverse Transcriptase (Invitrogen), 6 µg of random hexamer primers (Invitrogen), 0.67 mM dNTPs (Invitrogen), 40 U RNAse Out (Invitrogen), and First Strand Buffer (Invitrogen) in a 45 µL final reaction volume. The enzyme was then inactivated by incubating the reactions at 70°C for 15 min. The resulting cDNAs were tested in real-time PCR reactions to determine the absolute quantity of total and unspliced HIV-1 transcripts using the PCR master mix and cycling profile described above. Total initiated HIV-1 transcripts were amplified using primers HIVshortF and HIVshortR. PCR primers LA8 and LA9 [45] were modified to match the sequence of HIV_NL4-3_, and used to amplify unspliced HIV-1 transcripts. Dissociation curves were performed on all PCR reactions to determine the specificity of each PCR reaction. The absolute quantification data was analyzed using Sequence Detection Software v1.2.2 (Applied Biosystems).

## Results

### Tat-SF1 depletion does not affect T-Rex-293 cell viability

**Figure 1:**
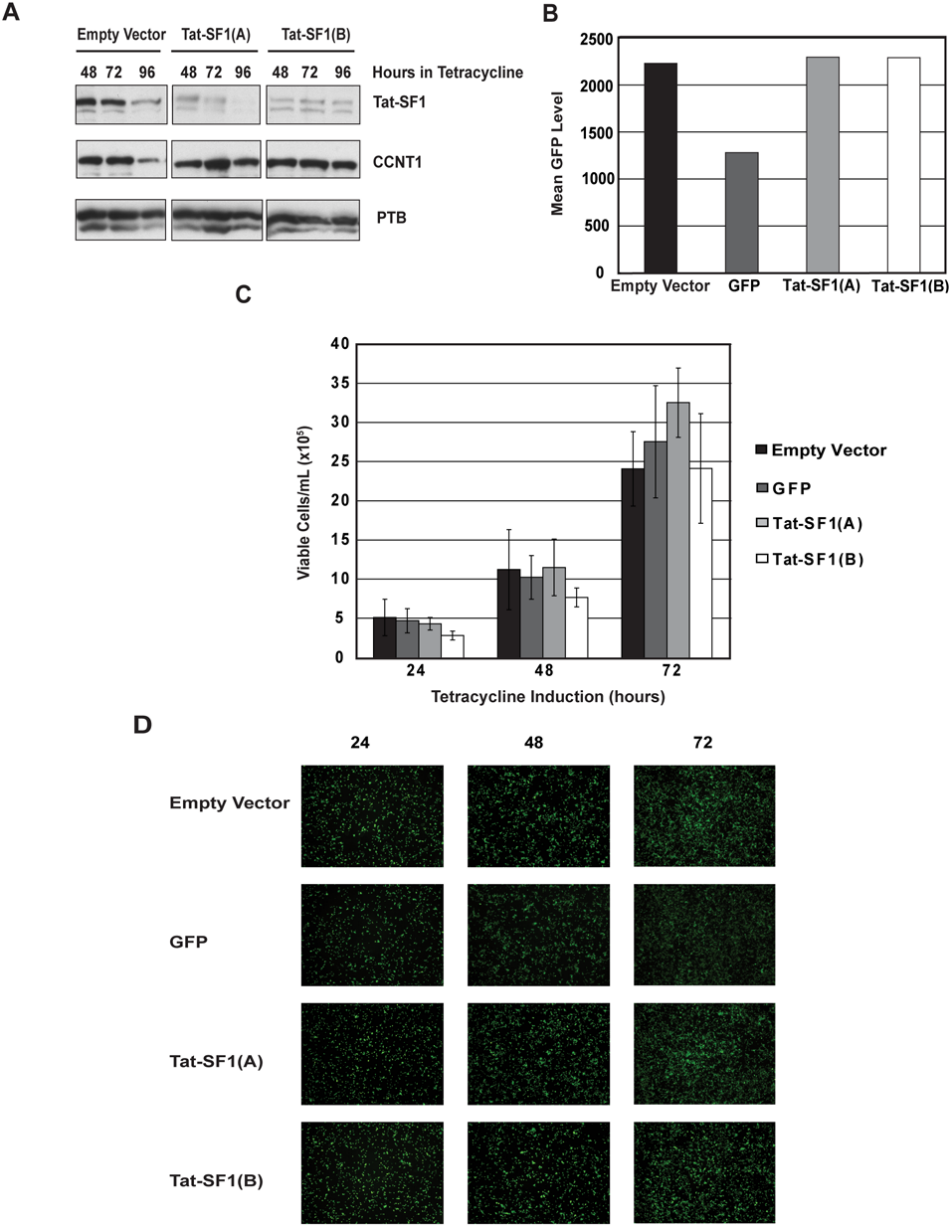
Tat-SF1 depletion does not affect T-Rex-293 cell viability. (A) Analysis of Tat-SF1 knockdown by Western blot. T-Rex-293 cells were induced with tetracycline and lysates were made at the time points indicated. Equal protein amounts were loaded for Western blot analysis with antibodies against Tat-SF1, CCNT1 and PTB. Lanes 1–3 are from cells that express an empty vector control and lanes 4–9 are from cells that express one of two unique shRNAs targeting Tat-SF1. (B) Analysis of GFP knockdown by flow cytometry. T-Rex-293 cells were induced with tetracycline for 72 hours, and resuspended cells were fixed with formaldehyde for flow cytometry analysis. The bar graph quantifies the mean GFP level of each sample. (C) Analysis of cell viability by trypan blue exclusion. Equivalent numbers of T-Rex-293 cells (expressing either an empty vector control, an shRNA targeting GFP, or one of two shRNAs targeting Tat-SF1) were induced with tetracycline for the times indicated. Viable cells per mL are reported as a means of three independent experiments. Error bars represent standard error. (D) Analysis of cell viability by fluorescence microscopy. Equal numbers of T-Rex-293 cells were induced with tetracycline for the times indicated and imaged with fluorescence microscopy.

To test the importance of Tat-SF1 in the HIV-1 life cycle *in vivo*, we used RNAi to specifically silence its expression. Four T-Rex-293 cell lines were constructed containing four unique shRNAs that targeted Tat-SF1, which were expressed from tetracycline-inducible promoters (see Materials and Methods). This system minimized continuous expression of the shRNAs until tetracycline was added to the media, thus reducing selection based on shRNA expression. All of the cell lines were tested for shRNA-mediated Tat-SF1 depletion, and the two most efficient, Tat-SF1(A) and Tat-SF1(B), were used in all subsequent experiments. In addition, two control cell lines were made that stably integrated either an empty vector (no shRNA) or an shRNA targeting GFP. Tetracycline concentration and induction time were optimized to achieve the maximum level of knockdown. Analysis of cell lysates from induced cells shows that both cell lines that contain an shRNA targeting Tat-SF1 have significantly lower Tat-SF1 protein levels compared to the empty vector control (Figure 1A). The knockdown of Tat-SF1 by either shRNA did not affect the levels of CCNT1, a direct binding partner of Tat-SF1, or the polypyrimidine tract binding protein (PTB), an unrelated splicing factor (Figure 1A). Flow cytometry demonstrated that the cell line containing an shRNA targeting GFP produced an active shRNA, as GFP levels were reduced by approximately 50% (Figure 1B) while Tat-SF1 levels were unchanged (data not shown). Tetracycline induction for 72 hours was chosen for the functional assays, although 96 hours also showed persistent knockdown.

We first sought to evaluate the effect of Tat-SF1 knockdown on T-Rex-293 cell viability. Trypan blue exclusion was used to quantify viability in the control and knockdown cells at 24, 48, and 72 hours post-tetracycline induction. Figure 1C shows the average number of viable cells per mL in each cell line. The number of viable cells per mL in the Tat-SF1 knockdown cell lines was not significantly different from the control cells at the end of the experiment. The Tat-SF1(B) shRNA shows slightly lower cell viability initially, however, this difference is not significant at later time points. The density of the Tat-SF1 knockdown cells was also indistinguishable from that of the control cells at each time point (Figure 1D). These data indicate that Tat-SF1 depletion did not affect T-Rex-293 cell viability, and therefore, we proceeded to assay the role of Tat-SF1 as a cellular cofactor of HIV.

### Tat-SF1 depletion inhibits HIV-1 infectivity

**Figure 2:**
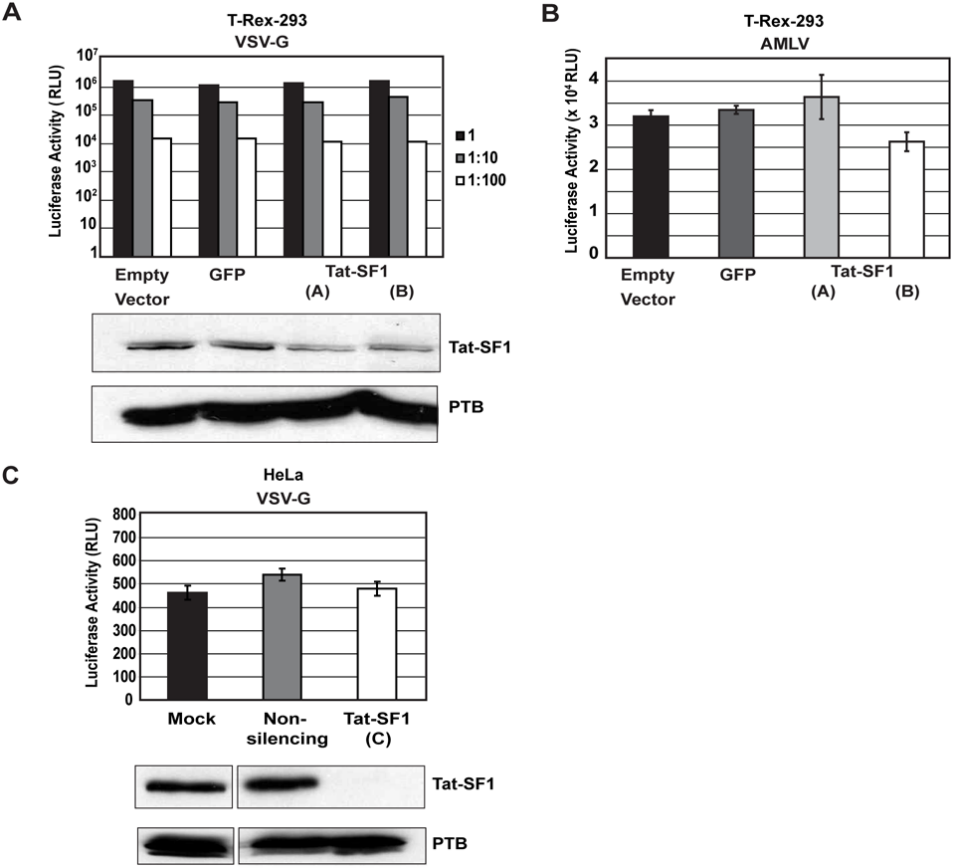
Tat-SF1 depletion does not affect pseudotyped HIV-1 replication. (A) VSV-G replication assay. Serial dilutions of pseudotyped virus (VSV-G envelope) were used in a single-round replication assay with T-Rex-293 cells expressing an empty vector, an shRNA targeting GFP, or shRNAs targeting Tat-SF1. A western blot confirming knockdown is shown below the chart. Luciferase values of the cell lysates were read after 24 hours. Luciferase values were background corrected and normalized to protein content. (B) AMLV replication assay. An AMLV-pseudotyped virus was used in the same type of experiment described in (A) except that lysates were harvested after 48 hours. Values reported are the means of triplicate wells. Error bars represent standard error. The western blot in (A) also corresponds to the cells used in this experiment. (C) VSV-G-replication assay. Pseudotyped virus was used on HeLa cells stably expressing a non-silencing shRNA or a third shRNA targeting Tat-SF1. Luciferase values of the cell lysates were read after 24 hours. Luciferase values were background corrected and normalized to protein content. Values are reported as the means of triplicate wells. Error bars represent standard error.

If Tat-SF1 was indeed required for Tat transactivation *in vivo*, then RNAi-mediated depletion of Tat-SF1 should decrease the ability of HIV-1 to replicate. To test this hypothesis, control and Tat-SF1 knockdown T-Rex-293 cells were infected with a vesicular stomatitis virus G-protein (VSV-G) pseudotyped virus in a single-round replication assay. Although this pseudotyped virus does not produce infectious progeny due to its lack of the HIV-1 envelope, we use the term replication here to refer to the reproduction of viral genomes. Serial dilutions of the viral stock were added to cells and lysates were assayed for luciferase activity, which indicates LTR-dependent transcription, processing, and expression. To control for any differences in cell number between control and knockdown cell lines, cells were imaged before and after infection to ensure equal confluency, and luciferase values were normalized to the protein content of the lysate. Surprisingly, both of the Tat-SF1 knockdown cell lines showed levels of HIV-1 replication that were no different than those in the empty vector and GFP control cells after 24 hours of infection (Figure 2A). This remained true even with a 1∶100 dilution of the viral stock. HIV-1 replication was also unaffected by Tat-SF1 knockdown after 48 hours of infection (data not shown).

It was possible that the VSV-G pseudotyped virus was infecting the cells so efficiently that any deficiency due to Tat-SF1 depletion was being masked. To address this possibility, we also assessed replication using an AMLV-pseudotyped virus, which infects target cells with a lower efficiency. Again, there was no significant difference in HIV-1 replication after Tat-SF1 knockdown (Figure 2B).

Earlier studies of Tat-SF1 function were performed in HeLa cells, so we next tested our hypothesis by infecting HeLa cells that stably expressed a third shRNA targeting Tat-SF1, Tat-SF1(C). Tat-SF1 depletion did not significantly affect cell viability in HeLa cells (data not shown). Again, replication in Tat-SF1 depleted cells was comparable to control cells (Figure 2C). This led us to conclude that either Tat-SF1 had a minimal effect on HIV-1 replication, or it affected a step not interrogated by the experiments with these pseudotyped viruses. Since Brass *et al.* identified Tat-SF1 as a host factor required for HIV-1 propagation using a replication-competent virus [37], we favored the latter explanation.

**Figure 3:**
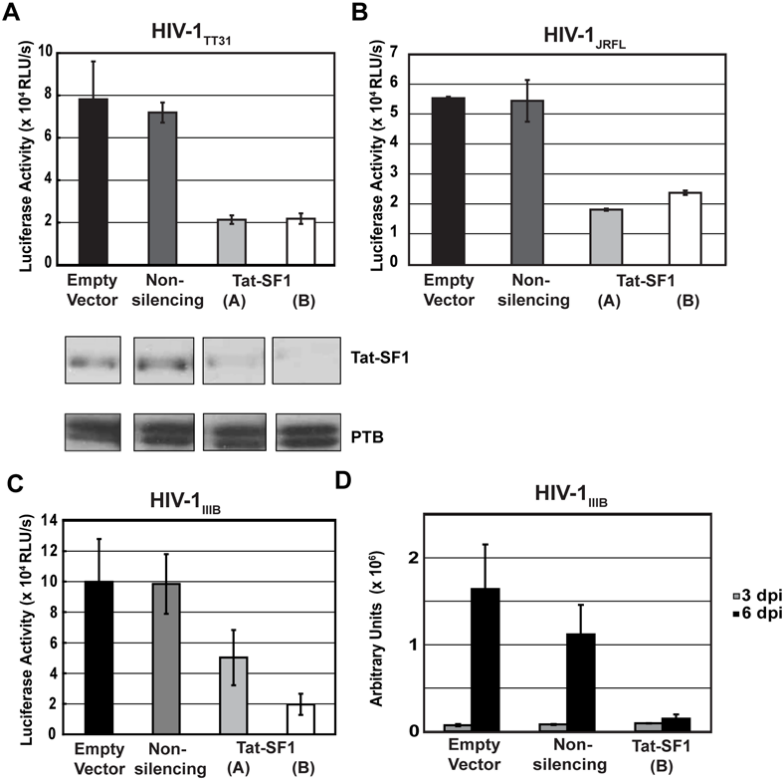
Tat-SF1 depletion inhibits HIV-1 infectivity. (A) TZM-bl assay using HIV-1_TT31_ . Replication-competent HIV-1_TT31_ was used to infect TZM-bl cells that were transiently transfected with either an empty vector, a non-silencing shRNA control, or shRNAs targeting Tat-SF1. A western blot confirming knockdown is shown below the chart. This western blot corresponds to cells used in Figures 3A–D. Luciferase values of the cell lysates were read 3 days after infection. Luciferase values were reported as the means of triplicate wells. Error bars represent standard error. (B) TZM-bl assay using HIV-1_JRFL_. Replication-competent HIV-1_JRFL_ was used in the same type of experiment described in (A). (C) TZM-bl assay using HIV-1_IIIB_. Replication-competent HIV-1_IIIB_ was used in the same type of experiment described in (A) and (B), except that cell lysates were read 6 days after infection. (D) Reverse transcriptase assay. Supernatants from infected cells in (C) were measured for reverse transcriptase activity 3 and 6 days after infection. Values from the phosphor screen image were reported as the means of triplicate wells. Error bars represent standard error.

In order to test if Tat-SF1 played a role in any step of the viral life cycle, we decided to assess the effect of Tat-SF1 depletion with a replication-competent virus. TZM-bl cells were used because they express CXCR4, CD4 and CCR5, and also contain integrated Tat-dependent beta-galactosidase and luciferase reporter genes. These cells are amenable to treatment with siRNAs [37] and have equal susceptibility to HIV-1 infection as compared to human peripheral blood mononuclear cells (PBMCs) [36], [46]. TZM-bl cells were transiently transfected with an empty vector, a non-silencing shRNA, or Tat-SF1 specific shRNAs. These cells were cotransfected with a GFP expressing plasmid to sort GFP-positive cells. GFP-positive transfectants were plated in 96-well plates and infected with one of two different replication-competent HIV-1 strains, HIV-1_TT31_ or HIV-1_JRFL_. HIV-1_JRFL_ is a laboratory adapted strain, whereas HIV-1_TT31_ encodes an envelope from an early-transmitted primary isolate [38]. After 3 days of infection, a luciferase assay was performed on the cell lysates. Upon Tat-SF1 depletion, the level of HIV-1 infection decreased approximately 3-fold compared to the control cells (Figures 3A and B). The decrease in infectivity was similar for both HIV-1 strains tested. A comparable decrease in infectivity after 6 days of infection was also observed (data not shown). Lysates from uninfected cells showed persistent knockdown of Tat-SF1 throughout the length of the experiment (data not shown).

As an independent, more direct measurement of viral infectivity, a third strain, HIV-1_IIIB_, was used to infect control and Tat-SF1 knockdown TZM-bl cells, and the viral supernatants from these cells were assayed for reverse transcriptase activity. Luciferase readings from the infected cell lysates recapitulated the decrease in infectivity upon Tat-SF1 depletion that was seen for the two HIV-1 strains used previously (Figure 3C). After 3 days of infection, both control and Tat-SF1 knockdown cells produced background levels of reverse transcriptase. After 6 days of infection, the most efficient knockdown cells, Tat-SF1(B), showed an inability to produce reverse transcriptase above background levels (Figure 3D).

Together, these data indicated that Tat-SF1 plays a positive role in regulating the lifecycle of HIV-1 at a step not interrogated by pseudotyped viruses. This conclusion was not easily reconciled with an effect on Tat transactivation.

### Tat-SF1 depletion does not affect Tat transactivation *in vivo*

**Figure 4:**
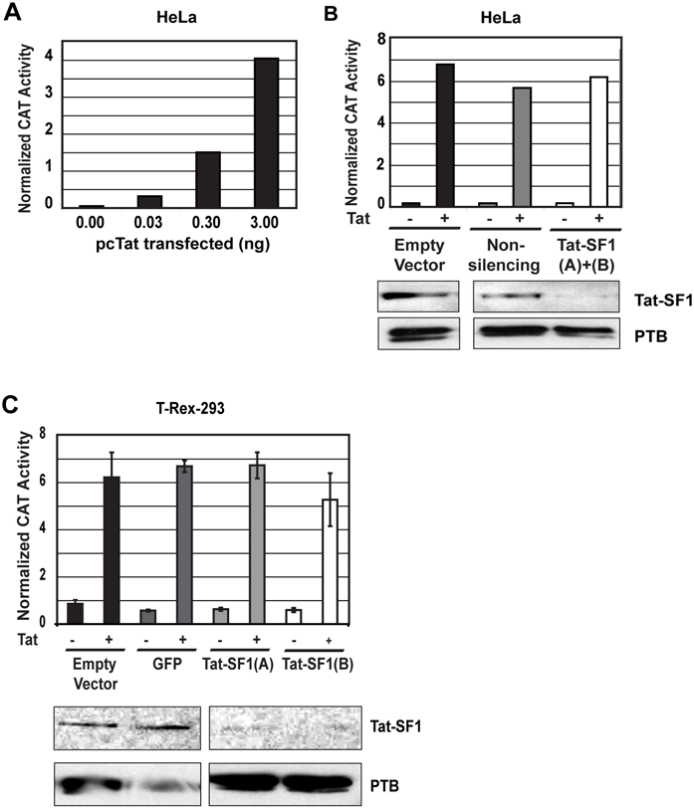
Tat-SF1 depletion does not affect basal or Tat-dependent transcription from the HIV-1 LTR *in vivo*. (A) Tat titration. HeLa cells were cotransfected with the indicated amount of pcTat, an HIV-1 CAT reporter, and an SV40-Luciferase reporter. Lysates were subjected to a CAT assay and normalized with values obtained from a luciferase assay 24 hours after cotransfection. All CAT and Luciferase values were background corrected. Values shown are the means of duplicate transfections. (B) Tat transactivation assay in HeLa cells. HeLa cells were transiently transfected with either an empty vector, a non-silencing shRNA, or two shRNAs targeting Tat-SF1. A western blot confirming knockdown is shown below the chart. Cells were cotransfected with the same reporters as in (A) plus 0.3 ng pcTat or an empty vector. All CAT and Luciferase values were background corrected, and CAT was normalized to Luciferase. Values shown are the means of duplicate transfections. Raw values are shown in Table 1, Exp. 1. (C) Tat transactivation in T-Rex-293 cells. T-Rex-293 cells harboring either an empty vector control, an shRNA targeting GFP, or one of two shRNAs targeting Tat-SF1 were induced with tetracycline for 48 hours. A western blot confirming knockdown is shown below the chart. Cells were cotransfected with the same reporters as in (A) and (B). All CAT and Luciferase values were background corrected, and CAT was normalized to Luciferase. Values shown are the means of triplicate transfections. Raw values are shown in Table 2. Error bars represent standard error.

Although Tat-SF1 has been implicated *in vitro* as a cofactor for the viral protein Tat, this had yet to be demonstrated *in vivo*. To examine Tat transactivation when Tat-SF1 was depleted, control and Tat-SF1 knockdown HeLa cell lines were transfected with a chloramphenicol acetyltransferase (CAT) reporter under the control of the HIV-1 LTR. This plasmid, along with an internal luciferase control for transfection efficiency, was cotransfected with either the Tat-expressing plasmid, pcTat [30], or an empty vector. The amount of pcTat transfected was experimentally determined so that Tat transactivation was in the linear range (Figure 4A). As expected, Tat stimulated transcription of the CAT reporter gene by approximately 40-fold in control cells (Figure 4B and Table 1). Surprisingly, similar levels of Tat transactivation were seen when Tat-SF1 was depleted. Furthermore, basal transcription of the LTR (in the absence of Tat) was unaffected by RNAi-mediated depletion of Tat-SF1. In addition, the same results were recapitulated in T-Rex-293 cells (Figure 4C and Table 2). These findings indicate that, contrary to the *in vitro* results, Tat-SF1 depletion did not affect basal or Tat-mediated transactivation of the HIV-1 LTR *in vivo*.

**Table 1:** Effect of Tat-SF1 depletion on basal and Tat-dependent HIV-1 transcription in HeLa cells*.

Reporter Gene	Exp.	Empty Vector			Non-silencing			Tat-SF1		
		−Tat	+Tat	Fold Change	−Tat	+Tat	Fold Change	−Tat	+Tat	Fold Change
CAT	**1**	269	13616	50.6	364	12554	34.5	301	11310	37.6
	**2**	110	2449	22.3	96	2511	26.2	65	2342	36.0
	**3**	722	10661	14.8	392	13500	34.4	437	10660	24.4
LUC	**1**	1652890	1997733	1.21	2034980	2203947	1.10	2034980	1846292	0.91
	**2**	190617	290975	1.53	138561	233280	1.68	172959	328568	1.90
	**3**	1781406	963590	0.54	1089682	691738	0.63	1316443	769614	0.58

* Values shown are the means of duplicate wells for 3 independent experiments. Row 1 is calculated from the same experiment shown in Figure 4B. CAT activity was the value of the slope of the linear function obtained by plotting cpm of acetylated chloramphenicol versus time. Luciferase activity of the same sample was measured with a luminometer. For both reporter assays, background values obtained from lysis buffer alone were subtracted from each sample.

**Table 2:** Effect of Tat-SF1 depletion on basal and Tat-dependent HIV-1 transcription in T-Rex-293 cells*.

Reporter Gene	Empty Vector			GFP			Tat-SF1(A)			Tat-SF1(B)		
	−Tat	+Tat	Fold Change	−Tat	+Tat	Fold Change	−Tat	+Tat	Fold Change	−Tat	+Tat	Fold Change
CAT	30	280	9.3	25	188	7.5	21	149	7.1	29	99	3.4
LUC	325516	491544	1.5	435065	281696	0.6	327725	218257	0.7	465079	198163	0.4

* Values shown are the means of triplicate wells, calculated from the same experiment shown in Figure 4C. CAT activity was the value of the slope of the linear function obtained by plotting cpm of acetylated chloramphenicol versus time. Luciferase activity of the same sample was measured with a luminometer. For both reporter assays, background values obtained from lysis buffer alone were subtracted from each sample.

### Tat-SF1 maintains the ratios of HIV-1 RNAs

**Figure 5:**
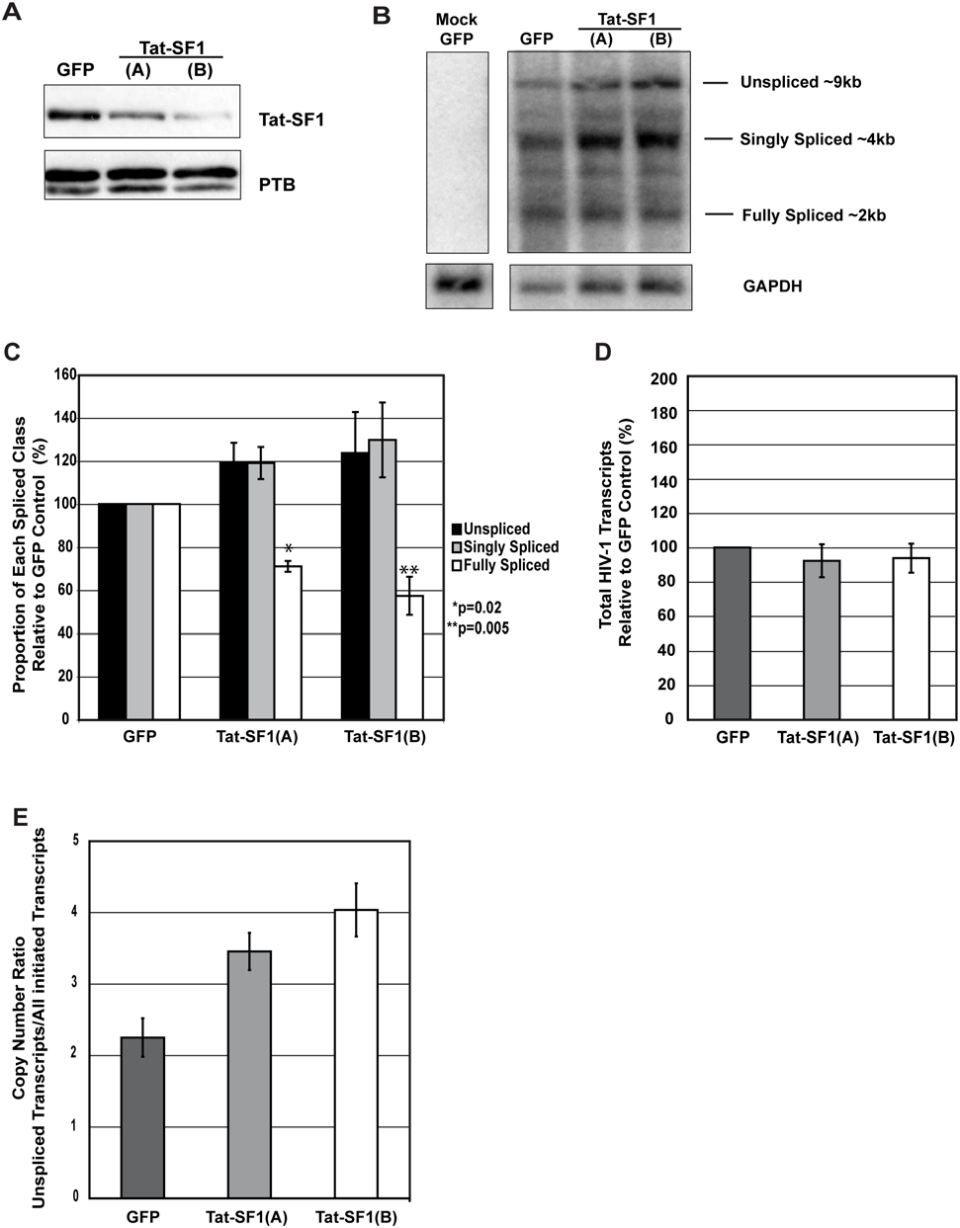
Tat-SF1 maintains the levels of unspliced and spliced HIV-1 RNAs. (A) Western blot analysis confirming knockdown in T-Rex-293 cells. (B) Representative Northern blot analysis of HIV-1 RNA classes. T-Rex-293 cells were transfected with pSG3ΔEnv 72 hours after tetracycline induction. At 48 hours post-transfection, total RNA was isolated for electrophoresis and Northern blotting. A DNA probe specific to the HIV-1 LTR detected the ∼9 kb unspliced, ∼4 kb singly spliced, and ∼2 kb fully spliced RNAs. Lane 1 contains RNA from mock-transfected GFP control cells, lane 2 from transfected GFP control cells, and lanes 3 and 4 from transfected Tat-SF1 shRNA cells. The lower panel shows the same membrane, stripped and reprobed for GAPDH. (C) Tat-SF1 depletion alters the levels of HIV-1 RNA classes. Values are reported as the mean proportion of each RNA class, relative to the GFP control cells from three independent Northern blot experiments. Error bars represent standard error. Statistically significant differences between GFP control and Tat-SF1 knockdown conditions are indicated with asterisks. (D) Tat-SF1 depletion does not alter total HIV-1 RNA levels. Levels of the 3 RNA classes quantified from triplicate Northern blots were totaled and normalized to GAPDH levels. Values are reported as the means relative to the GFP control cells from three independent experiments. Error bars represent standard error. (E) Tat-SF1 depletion results in an increase in unspliced HIV-1 transcripts. qRT-PCR was performed on the same RNA samples used for Northern blot experiments. The medians of triplicate amplifications (both unspliced products and all initiated HIV-1 transcripts) were calculated and means of unspliced transcripts/all initiated HIV-1 transcripts ratios from triplicate samples are reported. Error bars represent standard error.

Although Tat-SF1 depletion resulted in a decrease in HIV-1 infectivity, it did not affect Tat transactivation. Rather than having a role in transcription elongation of HIV-1 RNA, it seemed possible that Tat-SF1 could post-transcriptionally regulate viral gene expression. To test this hypothesis, we used Northern blots to analyze HIV-1 RNAs in control and knockdown cells. The pSG3ΔEnv plasmid was transfected into GFP control and Tat-SF1 knockdown T-Rex-293 cell lines, and total RNA was harvested 48 hours later. Hybridization was performed using an HIV-1 LTR-specific radiolabeled probe that detects all three classes of viral RNAs: unspliced (∼9 kb), singly spliced (∼4 kb), and fully spliced (∼2 kb). A western blot of T-Rex-293 cell lysates confirms efficient knockdown of Tat-SF1 (Figure 5A). Figure 5B shows the results of a representative Northern blot probing RNA from GFP control and Tat-SF1 knockdown cell lines 48 hours after transfection with the pSG3ΔEnv plasmid. In the mock transfected lane, no viral pre-mRNA signal is detected, indicating that the bands seen in the other lanes are HIV-1-specific. Quantification of each RNA class demonstrates that intron-containing unspliced and singly spliced transcripts were elevated and fully spliced transcripts were reduced when Tat-SF1 was depleted (Figure 5C). This corresponds to an unspliced/fully spliced ratio of ∼0.7 in GFP control cells and ∼1.6 in Tat-SF1 depleted cells. Similar changes in ratios were observed when another viral plasmid, pNL-Luc-HXB, was transfected into Tat-SF1 depleted T-Rex-293 cells (data not shown). These data further suggest a post-transcriptional role for Tat-SF1.

To test whether or not the total amount of HIV-1 transcripts was altered by Tat-SF1 depletion, total viral RNAs were quantified from triplicate Northern blot experiments and normalized to GAPDH. Indeed, total HIV-1 RNA levels were not significantly different between the control and Tat-SF1 knockdown cell lines (Figure 5D).

As an independent test of a change in the splicing pattern, the same RNAs used in the Northern blot experiments were analyzed by qRT-PCR. We attempted the specific amplification of the 9 kb, 4 kb and 2 kb classes using previously published oligos [47]. Nonetheless, separation the PCR products on an acrylamide gel revealed that, although the 9 kb primers produced one distinct band, the 4 kb and 2 kb primers produced multiple amplicons larger than the expected one (likely including unspliced RNAs). In addition, the qPCR melting curves indicated more than one product for the 4 kb amplification. In fact, careful inspection of the design of the oligos used in a previous report [47] suggests that the reported RNA-class specificity would be exceedingly difficult to achieve. Nevertheless, the quantification of the unspliced RNAs over all initiated transcripts confirmed that there was an increase in the 9 kb, unspliced transcripts upon Tat-SF1 knockdown, in concordance with the Northern blots (Figure 5E). This change in the viral splicing pattern suggests that Tat-SF1 plays a post-transcriptional role in regulating the ratio of unspliced to spliced HIV-1 RNAs *in vivo*.

## Discussion

The results presented here shed new light on the mechanism by which the human protein, Tat-SF1, functions in HIV-1 replication. The first important conclusion was that Tat-SF1 was not required for basal or Tat-dependent transcription from the HIV-1 LTR *in vivo*. This contradicts previously reported conclusions [16], [22]; however, careful reevaluation of the published data suggests concerns about these earlier inferences. First, as part of a multiprotein complex, immunodepletion of Tat-SF1 from nuclear extract may have resulted in co-depletion of other proteins essential for Tat transactivation, most notably, cyclin T1, which directly binds Tat-SF1. As a subunit of P-TEFb, co-depletion of cyclin T1 could certainly affect levels of transcription. Equally, RNAi-mediated depletion of CA150, which we previously hypothesized would be a Tat cofactor [17] and which is also known to interact with P-TEFb, did not show any effect on basal or Tat-dependent transcription from the HIV-1 LTR (data not shown). In light of these RNAi-mediated depletion experiments, it seems very possible that the identification of both Tat-SF1 and CA150 as Tat cofactors from Tat-affinity column experiments could be explained by their association with P-TEFb.

A previous report on Tat-SF1 function showed a small increase in Tat transactivation when Tat-SF1 was overexpressed [16]. As described earlier, this change was primarily due to a decrease in basal transcription, however, even this effect was not reproduced using a different promoter to drive expression of Tat-SF1 [22]. Finally, chromatin immunoprecipitation (ChIP) data indicated that Tat-SF1 was not present at an integrated HIV-1 LTR-driven reporter gene during Tat transactivation *in vivo*, while RNAPII and P-TEFb were [48]. This finding is difficult to reconcile with Tat-SF1 being a required Tat cofactor. Nonetheless, when a cell line harboring integrated proviral DNA was utilized for ChIP experiments, Tat-SF1 was detected at the promoter-proximal region [49]. In light of our data, we propose that the presence of viral splice sites or other elements in the HIV-1 transcripts explains the recruitment of Tat-SF1 to the proviral locus.

An alternative explanation for the different conclusions drawn from previous overexpression and our knockdown studies is that lack of functioning Tat-SF1 can be compensated by another cellular protein. SPT5 has also been reported to stimulate Tat transactivation when overexpressed [22] and inhibits transactivation when immunodepleted from nuclear extract [20] or depleted by RNAi [50]. CA150 did not compensate for Tat-SF1 depletion, as a double knockdown of both proteins did not affect HIV-1 transcription (data not shown). Our findings do not eliminate the possibility that Tat-SF1 plays some role in Tat transactivation, although it is not absolutely required. A similar caveat, which can be leveled against all silencing experiments, is that a knockdown is not tantamount to a knockout, and the remaining level of the silenced gene product is sufficient for activity. Nonetheless, depletion of Tat-SF1 did result in the reduced viral replication and altered ratios of viral transcripts, so the preponderance of the evidence strongly suggests that Tat-SF1 is not a required Tat stimulatory factor.

Another important and novel finding presented here is that Tat-SF1 depletion increased the ratio of unspliced to spliced viral transcripts. It should be noted that splicing patterns of HIV transcripts among different cell lines are highly uniform, suggesting that the tight regulation of these transcript ratios is crucial for the viral lifecycle [51], [52], [53].

Careful investigation of the changes in HIV-1 spliced ratios over time showed that accumulation of unspliced and singly spliced RNAs and reduction of fully spliced RNAs did not occur until 48 hours post-transfection (data not shown). These data highlight the importance of timing when analyzing HIV-1 spliced RNAs. Thus, we have focused our efforts for determining Tat-SF1's role in maintaining RNA ratios to later time points.

While an effect on transcript ratios can be explained by several different mechanisms, we propose that Tat-SF1 regulates alternative splicing of HIV-1 RNAs. This is consistent with previous data regarding the yeast protein CUS2, which is structurally similar to human Tat-SF1. Both proteins contain two RRMs in their N-terminus, but Tat-SF1 has a large acidic C-terminus that is absent in CUS2. The first RRMs of these proteins are 37% identical and 59% similar. The second RRMs are 30% identical and 56% similar. CUS2 associates with U2 snRNA in splicing extracts and co-immunoprecipitates PRP11, which is a subunit of SF3a. When anti-Tat-SF1 antibodies were used for immunoprecipitation, the human homologue of PRP11, SF3a66(SAP62), was also immunoprecipitated [27]. An effect of Tat-SF1 depletion on HIV-1 RNA ratios is also consistent with recent unpublished data from our laboratory that demonstrate that Tat-SF1 depletion changes the relative levels many alternatively spliced transcripts in human cells without affecting the total amount of these transcripts (H.B. Miller *et al.*, unpublished results). An effect on splicing could be direct, as proposed for CUS2. Thus, Tat-SF1 could help in the folding and activity of splicing factors such as U2 snRNAs, but it could also rework the folding of the HIV transcripts leading to efficient splicing. Tat-SF1 may also have an indirect effect on HIV-1 pre-mRNA splicing by regulating the processing of transcripts encoding other HIV dependency factors (HDFs) [37], [54], [55]. In fact, we analyzed the HDFs published by the Brass *et al.* and Konig *et al.* screens and found approximately 2-fold enrichment over chance alone in genes that also had evidence of Tat-SF1-regulated alternative splicing (p-values of 0.02 and 0.05, respectively) (H.B. Miller *et al*, unpublished results). Tat-SF1 could also be involved in virion packaging. The decrease in infectivity in Tat-SF1 depleted cells could be explained if Tat-SF1 was a chaperone protein, helping fold the viral pre-mRNA genome into productive virions. Such a role in viral RNA packaging would be consistent with Tat-SF1's role in influenza virus replication. Tat-SF1 was identified as a stimulatory host factor, possibly aiding in the formation of RNA-nucleoprotein complexes by acting as a molecular chaperone [56]. It remains to be seen whether Tat-SF1 binds HIV-1 pre-mRNA and helps package viral genomes into virions. An increase in the unspliced RNA upon Tat-SF1 knockdown could also be explained by Tat-SF1 having a role in RNA export from the nucleus, although this has not yet been tested. Additionally, Tat-SF1 may play a role in regulating the levels of unspliced and spliced HIV-1 RNAs by affecting their stability. The virus may rely on Tat-SF1 to destabilize unspliced RNAs in order to maintain an optimal ratio of unspliced and spliced RNAs.

Other studies that support a role for Tat-SF1 in HIV replication found that Tat-SF1 was upregulated in either HIV-1 gp120 stimulated primary T cells [57] or HIV-1 Nef overexpression in T cells [58]. The strongest validation of Tat-SF1's role in the HIV-1 lifecycle was revealed when it was identified in a large-scale, RNAi-based screen for HIV-dependency factors (HDFs) [37]. Despite the fact that these studies refer to Tat-SF1 as a Tat cofactor, their findings remain consistent with this protein being involved in the post-transcriptional control of HIV-1 RNA levels and further support the idea that Tat-SF1 depletion has a negative effect on the viral life cycle *in vivo*.
